# Ultrasound-Assisted Extraction of Anthocyanins from Haskap (*Lonicera caerulea* L.) Berries Using a Deep Eutectic Solvent (DES) DES Extraction of Anthocyanins from Haskap Berries

**DOI:** 10.17113/ftb.59.01.21.6869

**Published:** 2021-03

**Authors:** Amanda M.G. MacLean, Yasmini P.A. Silva, Guangling Jiao, Marianne S. Brooks

**Affiliations:** 1 Department of Process Engineering and Applied Science, Dalhousie University, 5273 DaCosta Row, PO Box 15000, Halifax, NS, B3H 4R2, Canada; 2 Faculty of Nutrition, Federal University of Goiás, Rua 227, qd. 68, Setor Leste Universitário, Goiânia, GO, 74605-080, Brazil

**Keywords:** anthocyanins, Box-Behnken design, deep eutectic solvent, green extraction, haskap

## Abstract

**Research background:**

Haskap berries are one of the richest natural sources of anthocyanins and their extracts can be used for nutraceuticals and functional food ingredients. Deep eutectic solvents (DES) comprising food-grade or generally recognized as safe (GRAS) components show promise as natural solvents, but have not been applied to haskap berries. Thus, the aim of this study is to investigate the extraction of anthocyanins from haskap berries using a DES consisting of citric acid and d-(+)-maltose.

**Experimental approach:**

The experimental approach used ultrasound-assisted extraction with a DES consisting of citric acid and d-(+)-maltose as the solvent to achieve a sustainable green extraction process. Response surface methodology (RSM) with a Box-Behnken experimental design was used to study the effect of varying the extraction temperature, time of extraction, *V*(solvent)/*m*(sample) ratio (mL/g) and the water volume fraction (%) in the DES on the total anthocyanin content (TAC) in the haskap berry extracts.

**Results and conclusions:**

Under the optimal extraction conditions (75 °C, 10 min, 50.4 mL/g and 90% water) a predicted TAC extraction on dry mass basis yielded 21.2 mg/g, with experimental error of 7.2%. The TAC yield and anthocyanin profiles were similar to those obtained with conventional organic solvents.

**Novelty and scientific contribution:**

This is the first study investigating the use of a food-grade DES comprising GRAS components for the extraction of anthocyanins from haskap berries. These results indicate that the studied DES (citric acid and d-(+)-maltose) is a suitable alternative solvent for extracting anthocyanins for food-grade applications.

## INTRODUCTION

Haskap (*Lonicera caerulea* L.) berries, a fruit native to Siberia and northeastern Asia, have recently entered the North American market (*1*) and are known for their high anthocyanin content (*2*). Anthocyanins are flavonoids associated with antioxidative, anti-inflammatory, and anticarcinogenic properties (*3*), and are highly desirable for their application as dietary supplements and natural colourants (*4*). Currently, they are extracted from natural sources using traditional solvents, such as methanol and ethanol (*5*).

The extraction of bioactive compounds from natural sources can be made more environmentally friendly with assisting technologies such as ultrasound to improve extraction efficiency (*6*), and the development of green solvents. Green solvents are alternatives to organic solvents, and may be non-petroleum derived, biodegradable, with low toxicity (*7*). Deep eutectic solvents (DES) can be produced by mixing two or more natural components capable of hydrogen bond interactions (*8*). DES have been used for extraction of several flavonoids, such as genistin, genistein and apigenin (*9*), icariin, catechin, (+)-catechin, quercetin, kaempferol, myricetin, quercetin-O-rhamnoside (*10*), rutin, α-mangostin and cryptotanshinone (*11*). Recently, the extraction of anthocyanins from grape skin (*12*, *13*), wine lees (*14*) and *Lycium ruthenicum* Murr. fruit (*15*) using various DES has been reported.

The objective of this study is to investigate the ultrasound-assisted extraction (UAE) of anthocyanins from haskap berries using a DES prepared of citric acid and d-(+)-maltose as an alternative, sustainable, food-grade solvent. The extraction parameters studies were: temperature, time, water volume fraction in the DES and solvent to sample ratio. A Box-Benhken design of experiments and response surface methodology (RSM) were used to maximize anthocyanin extraction with DES, and the anthocyanin profile with DES extract was compared with a conventional methanol extract.

## MATERIALS AND METHODS

### Chemicals

All chemicals used were of analytical and high performance liquid chromatography (HPLC) grade, and were purchased from Sigma-Aldrich, Merck (Oakville, Ontario, Canada). The anthocyanin HPLC standards included cyanidin-3,5-di-glucoside (C3,5GL), cyanidin-3-glucoside (C3GL), cyanidin-3-galactoside (C3GA), pelargonidin-3-glucoside (PL3GL) cyanidin-3-rutinoside (C3RT) and peonidin-3-O-glucoside (P3GL).

### Plant material

Frozen haskap berries (*Lonicera caerulea* L.) that were previously harvested and frozen at Northern Light Orchards (Saskatchewan, Canada) were used. Berries were freeze dried in a 4.5-litre bench-top freeze-dryer (FreeZone, Labconco, Kansas City, MO, USA) until constant mass, with final moisture content of 4.95% (fresh mass). Freeze-dried samples were wrapped in aluminium foil and kept in a desiccator at –18 °C. Immediately prior to extraction the samples were ground (Smartgrind, Black & Decker, Mississauga, ON, Canada) and sieved through a 0.5-mm (32 mesh) sieve.

### Solvent preparation

The deep eutectic solvent (DES) was prepared according to Jeong *et al.* (*13*), with modifications. Briefly, citric acid and d-(+)-maltose monohydrate were combined at a 4:1 molar ratio in powdered form, and then dissolved in Milli-Q water (MilliporeSigma, Merck, Oakville, Canada). The solution was placed on a rotary evaporator (HiTEC RE-51; Yamato Scientific America, Santa Clara, CA, USA) at 4.2 kPa and 50 °C for elimination of excess water (*16*). Prior to extraction, the appropriate volume of DES was diluted with Milli-Q water (Table 1). The solvent was always prepared and used for extraction in the same day.

**Table 1:** Box-Benhken experimental design with the levels for each investigated factor and resultant total anthocyanin content (TAC)

Run number	Temperature/°C (X_1_)	*t*/min (X_2_)	*φ*(water)/% (X_3_)	(*V*(solvent)/*m*(sample))/(mL/g) (X_4_)	*w*(TAC as C3GL)/(mg /g)
1	25	10	60	35	19.04
2	75	10	60	35	16.99
3	25	60	60	35	17.42
4	75	60	60	35	*
5	50	35	30	10	5.02
6	50	35	90	10	9.25
7	50	35	30	60	15.66
8	50	35	90	60	19.62
9	25	35	60	10	7.75
10	75	35	60	10	10.98
11	25	35	60	60	12.07
12	75	35	60	60	15.25
13	50	10	30	35	12.83
14	50	60	30	35	14.77
15	50	10	90	35	18.24
16	50	60	90	35	16.79
17	25	35	30	35	7.99
18	75	35	30	35	11.38
19	25	35	90	35	9.79
20	75	35	90	35	16.20
21	50	10	60	10	10.21
22	50	60	60	10	8.99
23	50	10	60	60	14.64
24	50	60	60	60	16.69
25	50	35	60	35	16.39
26	50	35	60	35	15.23
27	50	35	60	35	18.46
28	50	35	60	35	16.61
29	50	35	60	35	16.17
30	50	35	60	35	16.05
Optimized condition	75	10	90	50.4	19.8**

Run number is not indicative of experimental run order. *Data omitted; **Predicted yield: *w*(TAC as C3GL)=21.2 mg/g dm

### Experimental design

A Box-Behnken design for four factors (*17*) was used to determine the levels of the experimental parameters: extraction temperature in °C, time of extraction in min, water volume fraction in %, and solvent/sample ratio ((*V*/*m*)/(mL/g)) (Table 1). DES molar ratio was held constant at the optimized 4:1 citric acid/maltose ratio according to Jeong *et al.* (*13*).

### Ultrasound-assisted extraction of anthocyanins

Ultrasound-assisted extraction (UAE) was performed in an ultrasound water bath (Branson 2510R-DTH; Branson Ultrasonics Corp., Danbury, CT, USA) at 40 kHz and 100 W. Extractions were conducted in 5-mL glass tubes, where the appropriate mass of sample and volume of solvent were added according to the Box-Behnken design (Table 1). The tubes were covered with aluminium foil, vortexed for 10 s, and placed in the ultrasonic bath. Following extraction, samples were centrifuged (Sorvall RT1; Thermo Scientific, Madison, WI, USA) at 4 °C and 4000 rpm for 10 min. The supernatant was then filtered through a 0.45-µm syringe filter and immediately used for spectrophotometric analysis (*18*). For comparison with DES, methanol extraction was conducted using the following optimal parameters based on a similar study using a conventional organic solvent by Celli *et al.* (*18*): solvent/sample ratio 25:1 (mL/g), solvent composition 80% methanol solution and 0.5% formic acid, and extraction temperature 35 °C for 20 min.

### Determination of total monomeric anthocyanin content

Total anthocyanin content (TAC) was determined using the pH differential method (*19*) conducted in duplicate using a UV-Visible spectrophotometer (Genesys 10S UV-Vis; Thermo Scientific). The TAC of the extract was calculated using the following equations:

*A*=(*A*_510 nm at pH=1.0_–*A*_700 nm at pH=1.0_)–(*A*_510 nm at pH=4.5-_–*A*_700 nm at pH=4.5_) /1/

and

TAC)=(*A*·*M*·DF·10^3^)/(*ε*·*l*) /2/

where *A*_510 nm at pH=1.0_ and *A*_700 nm at pH=1.0_ are the absorbances of the solution adjusted to pH=1.0 with KCl and *A*_510 nm at pH=4.5_ and *A*_700 nm at pH=4.5_ are the absorbances of the solution adjusted to pH=4.5 with NaAc, each read at 510 and 700 nm, respectively, *A* is the absorbance value from Eq. 1, *M* is the molecular mass of 449.38 g/mol, DF is the dilution factor of 25, *ε* is molar absorption coefficient 26 900 M^-1^ cm^-1^ and *l* is path length (1 cm) (*3*). Results are expressed in mg of C3GL equivalents per g dry mass (dm) of haskap berries.

### RSM analysis and optimization

Minitab® (v. 17.3.1) was used for analysis (*20*). A polynomial model was fitted to the experimental results followed by backwards elimination to reduce the model to significant factors. The model was then analysed using analysis of variance (ANOVA) with a 0.05 level of significance. RSM was used to obtain optimized extraction conditions, and experiments were run to validate these results.

### Anthocyanin profiling by HPLC

Both DES and methanol extracts were injected on a Synergi 4 µm Max-RP C12 column, 80 A, 250 mm×4.6 mm (Phenomenex, Torrance, USA) reversed-phase column at 30 °C using diode array detection (DAD) at 520 nm (Agilent 1100 Series; Agilent Technologies, Hewlett-Packard, Waldbronn, Germany) (*21*). The elution was carried out at 0.8 mL/min flow rate using water/methanol (90:10) with 1.0% formic acid (A), and pure methanol with 0.1% formic acid (B) started at 10% B, increased to 20% B in 7 min, then 45% B in 13 min, up to 70% B in 5 min, ended at 100% B in 3 min and held for 3 min, and then returned to 10% B in 9 min. Chromatograms were acquired using ChemStation v. A.10.02 software (*22*). Peaks were identified and quantified by comparing their retention times obtained with those of the standards.

## RESULTS AND DISCUSSION

TAC yield using DES varied between 5.02 and 19.62 mg/g (Table 1). The final reduced model is shown in the following equation:

*w*(TAC)=–22.12+0.638·X_1_+0.319·X_2_+0.0748·X_3_+0.4554·X_4_–0.00378·X_1_^2^–0.00452·X_4_^2^–0.00605·X_1_·X_2_ /3/

where X_1_ is extraction temperature (°C), X_2_ is extraction time (min), X_3_ is volume fraction of water in DES and X_4_ is solvent/sample ratio (mL/g). ANOVA results are summarized in Table 2. It is evident that the lack-of-fit was not significant (p>0.05), indicating a good fit to the experimental data.

**Table 2:** ANOVA results of significant factors in quadratic model

Source	Df	Adj SS	Adj MS	F-value	p-value
Model	7	328.000	46.857	18.07	0.0001
Linear	4	218.185	54.546	21.04	<0.0001
Temperature/°C (X_1_)	1	11.740	11.740	4.53	0.047
*t*/min* (X_2_)	1	1.631	1.631	0.63	0.437
*φ*(water)/% (X_3_)	1	53.616	53.616	20.68	<0.0001
(*V*/*m*)/(mL/g) (X_4_)	1	145.078	145.078	55.96	<0.0001
Quadratic	2	86.503	43.251	16.68	<0.0001
X_1_^2^	1	27.051	27.051	10.43	0.004
X_4_^2^	1	48.513	48.513	18.71	<0.0001
Two-way interaction	1	20.278	20.278	7.82	0.012
X_1_·X_2_	1	20.278	20.278	7.82	0.012
Error	19	49.256	2.592		
Lack-of-fit	14	43.460	3.104	2.68	0.141
Pure error	5	5.796	1.159		
Total	26	377.255			

R^2^=86.94%, R^2^(adj)=82.13%, R^2^(pred)=69.34%; *In the final model, while time as a single factor is not significant (p>0.05), it is significant in the 2-way interaction and is therefore included

**Fig. 1:**
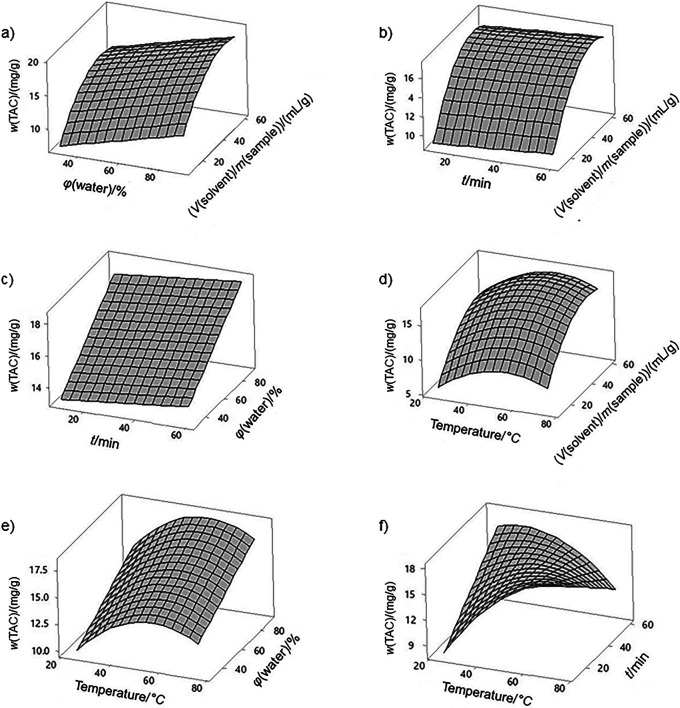
Surface plots of total anthocyanin content (TAC) extraction: a) *φ*(water)/% × (*V*(solvent)/*m*(sample))/(mL/g), b) *t*/min × (*V*(solvent)/*m*(sample))/(mL/g), c) *t*/min × (*φ*(water)/%, d) temperature/°C × (*V*(solvent)/*m*(sample))/(mL/g), e) temperature/°C × (*φ*(water)/%, and f) temperature/°C × *t*/min

Surface plots are shown in Fig. 1. Fig. 1f shows that high yields are obtained at either a combination of high temperature for short time or at lower temperature for longer extraction time. The optimized extraction conditions are X_1_=75 °C, X_2_=10 min, X_3_=90% water in DES and X_4_=50.4 mL/g, with a predicted TAC response on dry mass basis of 21.3 mg/g. Validation experiments at these conditions resulted in TAC of 19.8 mg/g. Although anthocyanin degradation is reported at high temperatures (*23*), the optimal conditions were at the highest temperature (75 °C). Temperature generally contributes to higher extraction yields due to enhanced mass transfer mechanisms (*24*). In the present study, the chemical environment of the DES system may also have had a positive influence in the thermal stability of the extracted compounds. The main degradation mechanisms related to thermal processing of anthocyanins include oxidation, cleavage of covalent bonds or enhanced oxidation reactions (*25*), and these processes are influenced by several factors, such as chemical structure of anthocyanins, acylation of the molecule, pH, light, oxygen, temperature, exposure time, presence/activity of enzyme polyphenol oxidase, and presence of other substances (*26*-*28*). During thermal processing, these factors may act synergistically, contributing to a higher stability of the bioactive compounds. In the present study, factors such as thermal inactivation of the enzyme polyphenol oxidase and the pH of the DES system may have contributed to higher stability at high temperature. Furthermore, it is possible that the DES could have caused acylation of the anthocyanin molecule, another factor that improves anthocyanin thermal stability (*25*, *28*). Bubalo *et al*. (*29*) reported higher extraction of anthocyanins in organic acid-based DES than in DES of lower acidity, achieving optimal UAE yield at 65 °C for 50 min. The short extraction time needed to achieve optimal yield (10 min) is a positive aspect of the process, showing that the DES system can easily access the intracellular structure to promote extraction. Furthermore, the short extraction time would reduce thermal degradation.

The highest water volume fraction (%) in DES that resulted in the optimum TAC yield was 90%. To ensure that pure water (without DES) was not more efficient, extraction was performed under optimal conditions using 100% water as the solvent, resulting in a TAC of 17.3 mg/g, significantly lower than optimal DES yield. The increased water volume fraction likely increased the extraction efficiency by decreasing the viscosity of the pure DES, allowing higher access to the plant intracellular structure (*24*) and higher mass transfer rates (*30*). This may also indicate a high polarity of the extracted anthocyanins, as more polar anthocyanins are extracted better with DES containing higher water volume fraction (*16*, *29*). The high water volume fraction in the solvent has also a positive economic benefit, as it decreases the overall cost of the solvent.

The use of DES to extract anthocyanins from other plant materials has been reported by other researchers. For example, Jeong *et al.* (*13*) evaluated the same DES system used in the present work and found it to be an efficient method for extracting anthocyanins from grape skins, when compared with traditional solvents including water, methanol, 80% aqueous methanol, ethanol and 70% aqueous ethanol. Radošević *et al.* (*31*) and Bosiljkov *et al.* (*14*) both used DES made of a combination of choline chloride with malic acid, and found this DES to be more efficient for extraction of anthocyanins than 70% methanol and acidified ethanol, respectively. Sang *et al.* (*15*) used a DES of choline chloride and 1,2-propandiol and found increased anthocyanin extraction compared with acidified methanol. Given these results, DES are an effective alternative to methanol for extracting anthocyanins. Previous work conducted by our research group using the same haskap berries (*18*) showed that conventional extraction using acidified aqueous ethanol (80%) resulted in a TAC yield of 22.73 mg/g. This is comparable to the TAC yield of 19.8 mg/g from the present study, indicating that similar TAC yields are achieved with this DES system.

**Fig. 2:**
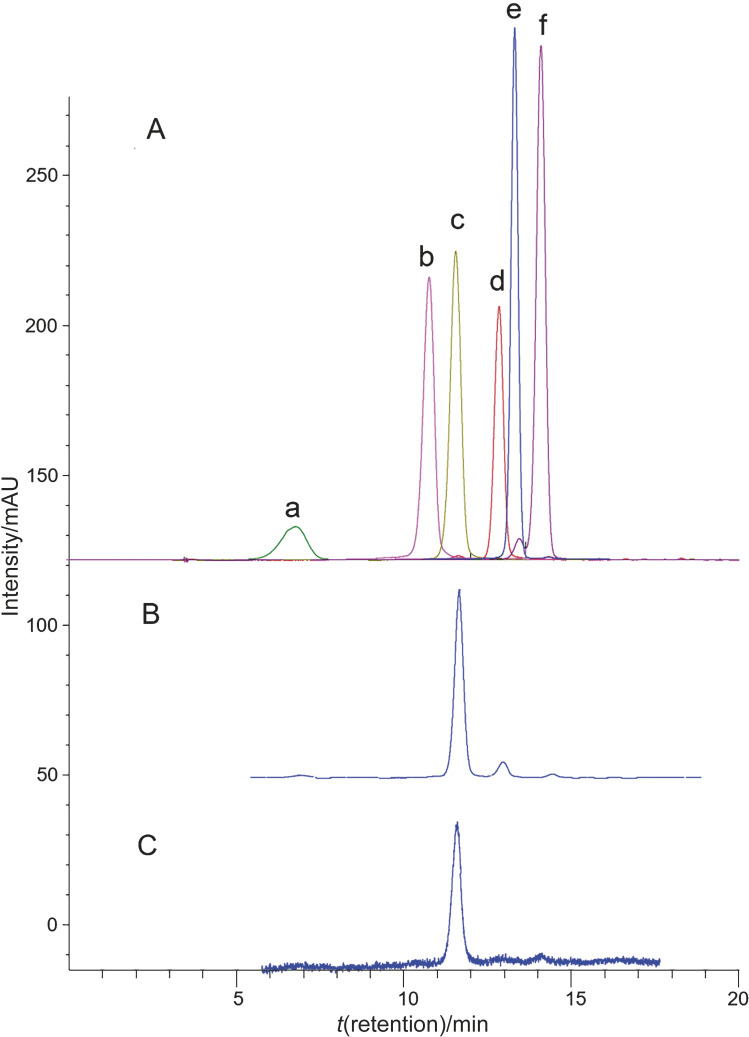
Anthocyanin profile from HPLC-DAD analysis: A=anthocyanin standards (a= cyanidin-3,5-di-glucoside (C3,5GL), b=cyanidin-3-galactoside (C3GA), c=cyanidin-3-glucoside (C3GL), d=cyanidin-3-rutinoside (C3RT), e=pelargonidin-3-glucoside (PL3GL), f=peonidin-3-O-glucoside (P3GL)), B=methanol extract, C=deep eutectic solvent (DES) extract

The results from HPLC-DAD analysis of DES and methanol extracts from haskap berries indicate that similar anthocyanin profiles were obtained at 520 nm (Fig. 2). The major component of both extracts was identified as C3GL, which comprises over 80% total anthocyanins, along with other small amounts of C3RT and P3GL. These data are in accordance with the anthocyanin profile of haskap berries that was reported in a previous study (*3*). The chromatogram in Fig. 2 suggests that DES had the same anthocyanin-extracting capacity from haskap berries as the conventional acidified methanol.

These results indicate that this DES system is an effective alternative to organic solvents for sustainable anthocyanin extraction. As citric acid and d-(+)-maltose are considered GRAS, this has important implications for the application of DES solvents that are GRAS in the food industry. For example, DES solvents comprising GRAS components could be used to extract bioactive compounds from food processing by-products to increase the sustainability of food production. Apart from that, the DES extracts could be used as novel functional food ingredients and incorporated directly into food products without post-extraction purification, where the DES components may confer additional benefits to the final product. For example, citric acid is an antioxidant and could add to the antioxidant activity of the bioactive compounds in the extract and the final food product.

## CONCLUSIONS

This is the first investigation of anthocyanin extraction from haskap berries using a deep eutectic solvent (DES). Optimized conditions for ultrasound-assisted extraction of total anthocyanins using a citric acid/d-(+)-maltose DES were: extraction temperature 75 °C, extraction time 10 min, solvent/sample ratio 50.4 mL/g and volume fraction of water in DES 90%. The maximum total anthocyanin yield on dry mass basis was 19.8 mg/g dm, and similar anthocyanin HPLC profiles were obtained with DES and methanol extracts. These results indicate that this DES system is an effective green alternative to organic solvents for sustainable anthocyanin extraction. The prospects for DES extraction in the food industry are promising as they could increase the sustainability of the food industry by utilizing processing by-products and replacing organic solvents with renewable GRAS components. Furthermore, DES extracts using GRAS components could be used as novel functional food ingredients. Future studies should investigate possible chemical interactions between the DES system and the anthocyanins, and the stability of the extracts.
